# Chemoenzymatic synthesis with the *Pasteurella* hyaluronan synthase; production of a multitude of defined authentic, derivatized, and analog polymers

**DOI:** 10.1002/pgr2.70000

**Published:** 2024-10-06

**Authors:** Paul L. DeAngelis

**Affiliations:** Department of Biochemistry and Physiology, University of Oklahoma Health Sciences Center, Oklahoma City, Oklahoma, USA

**Keywords:** defined oligosaccharides, glycosaminoglycans, hyaluronan, probes, quasi-monodisperse polysaccharides, UDP-sugars

## Abstract

Hyaluronan (HA; [-3-GlcNAc-1-beta-4-GlcA-1-beta]*_n_*), an essential matrix polysaccharide of vertebrates and the molecular camouflage coating in certain pathogens, is polymerized by “HA synthase” (HAS) enzymes. Three HAS classes have been identified with biotechnological utility, but only the Class II PmHAS from *Pasteurella multocida* Type A has been useful for preparation of very defined HA polymers in vitro. Two general chemoenzymatic strategies with different size products are possible: (1) repetitive step-wise extension reactions by sequential addition of a single monosaccharide from a donor UDP-sugar onto an acceptor (or “primer”) comprised of a short glycosaminoglycan chain (e.g., HA di-, tri- or tetrasaccharide) or an artificial glucuronide yielding homogeneous oligosaccharides in the range of 2 to ~20 monosaccharide units (*n* = 1 to ~10), or (2) “one-pot” polymerization reactions employing acceptor-mediated synchronization with stoichiometric size control yielding quasi-monodisperse (i.e., polydispersity approaching 1; very narrow size distributions) polysaccharides in the range of ~7 kDa to ~2 MDa (*n* = ~17 to 5000). In either strategy, acceptors containing non-carbohydrate functionalities (e.g., biotin, fluorophores, amines) can add useful moieties to the reducing termini of HA chains at 100% efficiency. As a further structural diversification, PmHAS can utilize a variety of unnatural UDP-sugar analogs thus adding novel groups (e.g., trifluoroacetyl, alkyne, azide, sulfhydryl) along the HA backbone and/or at its nonreducing terminus. This review discusses the current understanding and recent advances in HA chemoenzymatic synthesis methods using PmHAS. This powerful toolbox has potential for creation of a multitude of HA-based probes, therapeutics, drug conjugates, coatings, and biomaterials.

## HYALURONAN, A MULTIPURPOSE CARBOHYDRATE WITH MANY ROLES

Hyaluronan (hyaluronic acid; HA; [-3-GlcNAc-1-beta-4-GlcA-1-beta-]_n_) is an extracellular polysaccharide essential for the matrix formation in vertebrates and encapsulation in certain pathogenic microbes and virally infected hosts.^1–3^ Depending on the organism and exact tissue, HA chains ranging in molecular weight (MW) from ~10 kDa to 10 MDa (~25–25,000 sugar repeats) are synthesized and exported.^4–8^ After biosynthesis, fragmentation of HA by hyaluronidases (HAases) and/or reactive oxidative species results in a spectrum of smaller sizes.^9^ The field has recognized that the HA chain size can determine its solution behavior as well as its biological properties (reviewed in [9–14]), but there is much more work to be done as the polymer’s roles can differ among various organisms, tissues, and developmental stage. Perturbation of the native HA size distribution is observed in diseases such as cancer, chronic inflammation, and arthritis. Overall, there is a wide spectrum of HA chain sizes permeating the human body, but it is currently challenging to comprehend the full extent of this polymer’s roles and impacts.

Addition of exogenous HA to cell cultures, tissues, and organisms can modulate their behavior and/or health. Therefore, it is very important to have HA of defined size ranges for these experiments, however, this need is confounded by the fact that natural HA preparations have very wide size distributions, termed polydisperse. Tagged HA-probes and HA analogs of known structure are critical for understanding complex aspects of metabolism, transport, adhesion, organogenesis, and homeostasis. Defined and selective therapeutics are paramount for regulatory approvals and predictable patient therapies.

There are many methods to make various HA polymers (reviewed in [15, 16]), but some are more controllable and employ “green” routes minimizing the use of solvents, toxic reagents, and nonrenewable resources. One of these exemplary methods, chemoenzymatic synthesis, employs enzymes to catalyze selective controllable reactions to produce desirable targets. Twenty-five years ago, a microbial glycosyltransferase, PmHAS, that polymerizes the stealthy HA camouflage coating of a virulent multi-host pathogen was discovered. In the last two decades, this enzyme has been transmuted into various elegant catalysts with the potential to help elucidate the secrets of HA, the “wonder goo”.

## OVERVIEW OF HA SYNTHASES; THREE DISTINCT CLASSES

HA synthases (HASs) are amazing enzymes that co-polymerize the monosaccharides from two different UDP-activated sugars (UDP-GlcA and UDP-GlcNAc) with two different glycosidic linkages.^8^ In fact, these glycosyltransferases (GTs) were the first examples discovered that broke the early glycobiology dogma of “one enzyme, one linkage”.^17^ Three distinct varieties of bifunctional glycosyltransferases (GTs) with different architectures and reaction modes exist^18–21^; an updated HAS classification system was recently proposed.^8^

Class I membrane-integrated HASs employ a processive chain elongation mechanism and translocate HA directly across the plasma membrane. These enzymes contain a single GT family-2^22^ module that adds both monosaccharides to the nascent chain that then uses its intrinsic membrane-spanning channel to secrete the HA directly out of the cell. The processivity (i.e., retaining a HA chain throughout biosynthesis until completion) is theoretically due to the GT active site being in close proximity to or engaged with the channel. It is very interesting that the two subclasses of Class I catalyst exist which add incoming sugars to the growing chains with opposite directionality. The enzymes of Group A and C *Streptococcus* bacteria (SpHAS and SeHAS, respectively) add new sugars to the reducing terminus (Class I-R) while those of vertebrates (HAS1, 2, or 3) and an algal virus (CvHAS) add incoming sugars to the nonreducing terminus (Class I-NR). Some basic mechanistic details for HA initiation and polymerization by the viral CvHAS (& probably vertebrates) have been revealed with recent cryo-electron microscopy studies,^23^ but the streptococcal HAS remains a mystery. For the mouse and human HASs, the three isozymes synthesize polymers with different MW ranges^5^ suggesting a major role in the HA size distribution present in various tissues, potentially altering this carbohydrate’s bioactivity and behavior as mentioned earlier.

In contrast, PmHAS, the Class II HAS of *Pasteurella multocida* Carter Type A and related allies possesses a completely different architecture from the Class I enzymes.^24^ In fact, the PmHAS amino acid sequence is so unlike the Class I HASs that a totally independent gene isolation was required. An agnostic transposon insertional mutagenesis and visual screening approach was required to locate the desired target in HA-encapsulated *Pasteurella*^25^ because typical DNA-based hybridization methodologies using the streptococcal *HasA*/SpHAS sequence, the first HAS to be identified from any source,^26^ was unsuccessful. PmHAS is a peripheral membrane-associated enzyme with a nonprocessive, nonreducing end elongation mechanism using two independent GT-2 modules (one for each type of monosaccharide)^24^ and requires a separate secretion system to facilitate HA export (reviewed in [27]).

No three-dimensional structure for PmHAS has been determined, but a crystal structure for the *E. coli* K4 chondroitin synthase^28^ supports the overall two GT-2 domain model in a single PmHAS polypeptide. The PmHAS protein functionally acts as a monomer based on radiation inactivation studies^29^ simplifying its use as a catalyst in vitro.

The overall co-polymerization reaction catalyzed by PmHAS is shown in Equation 1. The enzyme requires a divalent metal ion; manganese appears to be the best for the overall reaction, but other metals like magnesium and cobalt can substitute. It was noteworthy that the two component glycosyltransferases of PmHAS had different metal preferences in vitro.^30^

(1) $$n\;\text{UDP}-\text{GlcNAc}+n\;\text{UDP}-\text{GlcA}\overset{PmHAS}{\underset{{\text{Mn}}^{2+}}{\to }}2n\;\text{UDP}+{\left[\text{GlcNAc}-\text{GlcA}\right]}_{n}=\text{HA}$$

The relatively independent GT modules allow for new synthesis strategies well beyond current Class I catalytic capabilities and may be part of the underlying reason for tolerance of sugar analogs as discussed later. A recombinant *E. coli*-derived truncated version of PmHAS with the first 703 amino acid residues of the native 972-residue sequence has proven useful; this catalyst is very soluble (~50 mg/mL) and produced at higher expression levels (~30 mg/L culture) when the hypothetical membrane docking domain is deleted.^24^ General protocols for PmHAS (and other *Pasteurella* GAG synthases) production and handling have been reported.^31^ The truncated PmHAS^1-703^ construct does not require tagging for purification, but the use of hisidine_6_-tagged versions can also be used.^32^ Using a combined error-prone PCR, modeling and directed evolution approach, it was reported that a PmHAS variant with several point mutations near the *N*-terminus yielded higher MW HA species in vitro than the wild-type sequence and it was speculated that added the polypeptide flexibility in that area was potentially responsible.^33^

The use of ethylene glycol^24^ or potassium chloride^34^ to substantially enhance and/or stabilize PmHAS activity has been reported, but reaction buffers could be improved. Another very unique and favorable feature of PmHAS is its relative insensitivity to the UDP byproduct released during HA polymerization (~60% inhibited with 15mM UDP and 1mM UDP-sugars). In contrast, most Class I enzyme reactions are significantly slowed (>90% when the UDP levels approach 0.5 mM).^35^ Therefore, very high UDP-sugar donor incorporation efficiencies can be routinely obtained with PmHAS and authentic HA precursors.

The focus of this review is the in vitro chemoenzymatic utility of PmHAS and its mutants to produce a multitude of very defined HA or HA-like polymers spanning a wide chain size spectrum. Two basic chemoenzymatic strategies and their permutations allow the design and controlled construction of polymers with either natural or artificial structures.

### Method 1: Synthetic HA oligosaccharides via stepwise addition reactions

Enzymatic cleavage of HA in vitro can yield HA oligosaccharide mixtures, thus requiring purification steps to isolate the more desirable defined targets.^36,37^ Typically, extensive digests are performed in vitro using bacterial GAG lyases, testicular HAase, or leech HAase for products with 1–3 HA disaccharide repeats while more limited digests are employed for larger oligosaccharide targets (and over-digestion is possible). The use of reaction conditions with copper ion can modulate testicular HAase to assist the preparation of larger oligosaccharides.^38^

Fortunately, the preparation of any specific size oligosaccharide in the range from 2 to ~20 monosaccharide units is facilitated by the availability of the non-processive Class II PmHAS that can elongate exogenously added acceptor molecules (Equation 2); no Class I enzyme is currently suitable for this application thus far.^20^ The acceptor is a surrogate for the in vivo initial primer, a KDO-lipid (reviewed in [27]), or for the nascent HA chain during polymerization. Short HA tetrasaccharides (*n* = 2) isolated from hyaluronidase digests were initially employed as the acceptor or primer,^35^ but later synthetic HA trisaccharides,^39^ synthetic glucuronides,^32,39,40^ or chondroitin oligosaccharides (the GalNAc C4-epimer of HA)^41^ were shown to be useful (more discussion later).


(2) $$n\;\text{UDP}-\text{GlcNAc}+n\;\text{UDP}-\text{GlcA}+\text{acceptor}\to 2n\;\text{UDP}+{\left[\text{GlcNAc}-\text{GlcA}\right]}_{n}-\text{acceptor}$$

HA oligosaccharide synthesis is possible with either soluble or bead-immobilized recombinant PmHAS-derived catalysts^20,35^ (Table 1). The former method employs PmHAS to add the monosaccharide from a single UDP-sugar donor (either UDP-GlcA or UDP-GlcNAc) to a suitable acceptor (a GlcNAc- or GlcA-terminated HA oligosaccharide, respectively). When using a soluble bifunctional PmHAS, before the next sugar addition step using the alternative donor to form the HA repeat, the HA oligosaccharide intermediate must be purified to avoid runaway polymerization reactions that would quickly occur if both UDP-sugar donors are present simultaneously in a reaction.

**FIGURE 1:**
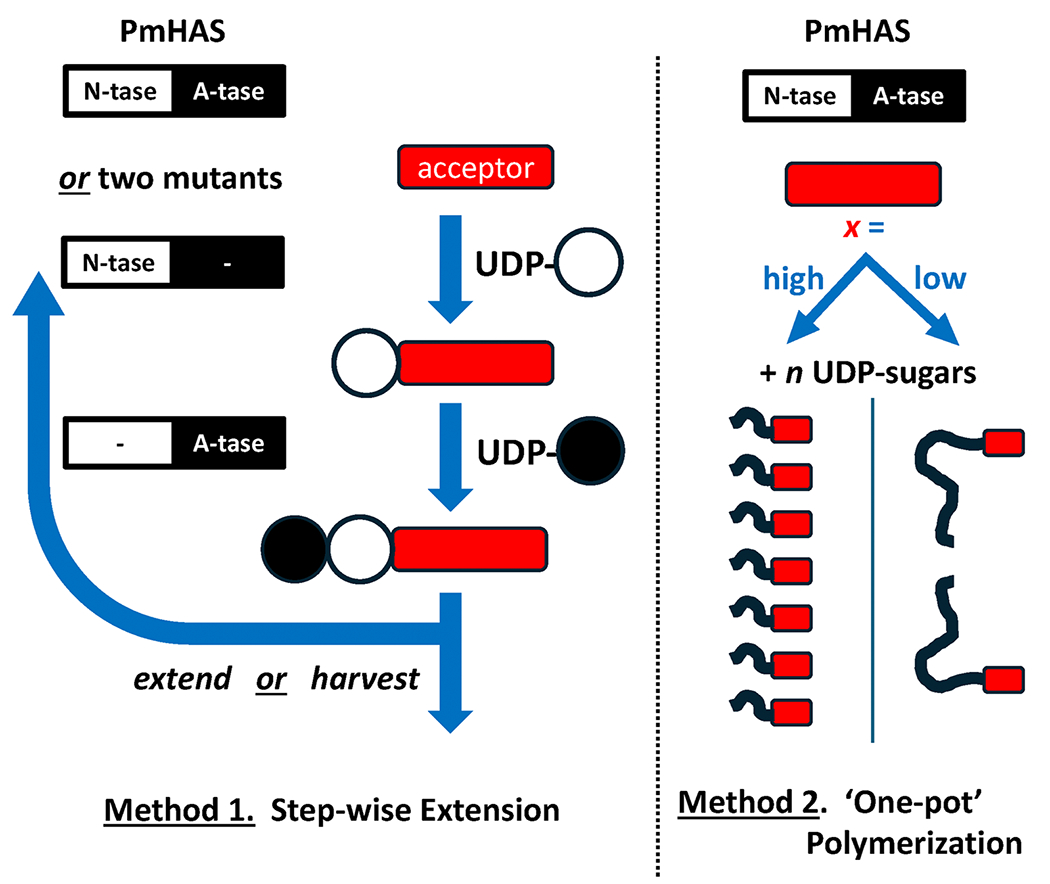
General PmHAS-based *in vitro* chemoenzymatic synthesis strategies for defined HA and HA-like polymer targets in a wide size range. Two basic methods are employed for either shorter or longer MW HA targets. Method 1: Step-wise elongation, is used to build a defined HA oligosaccharide in a range of 2 to ~20 monosaccharide units by extending an acceptor (*red*) with a UDP-sugar donor. If bifunctional PmHAS is used, then the sugar product must be purified after each single sugar extension step to prevent runaway polymerization when the next UDP-sugar species is added. However, if the enzyme is mutated (converting DXD motif into NXN) into a pair of its component glycosyltransferases, a GlcNAc-transferase (N-tase), and a GlcA-transferase (A-tase), and then these single-action catalysts are immobilized to allow separate extension reactions, then no intermediate purifications are required between extension steps. Method 2: “One-pot” synchronized polymerization, builds a quasi-monodisperse HA preparation in the range from ~35 to ~10,000 monosaccharide units by extending an acceptor of three to four monosaccharide units (*note*: simple mono- or disaccharides are not appropriate for synchronization) with UDP-sugar donors in a distributive fashion. In these reactions, all of the acceptors are elongated in parallel thus the HA product chains have very similar MWs. The particular chain size selected for a given reaction is controlled by the donor:acceptor ratio; basically, for a given amount of UDP-sugars, the final polymer size is controlled by *x*, the amount of acceptor as in Equation 5. The chemical complexity of the polymer products in Methods 1 and 2 may also be expanded as depicted in Figure 3 and Table 2.

**FIGURE 2:**
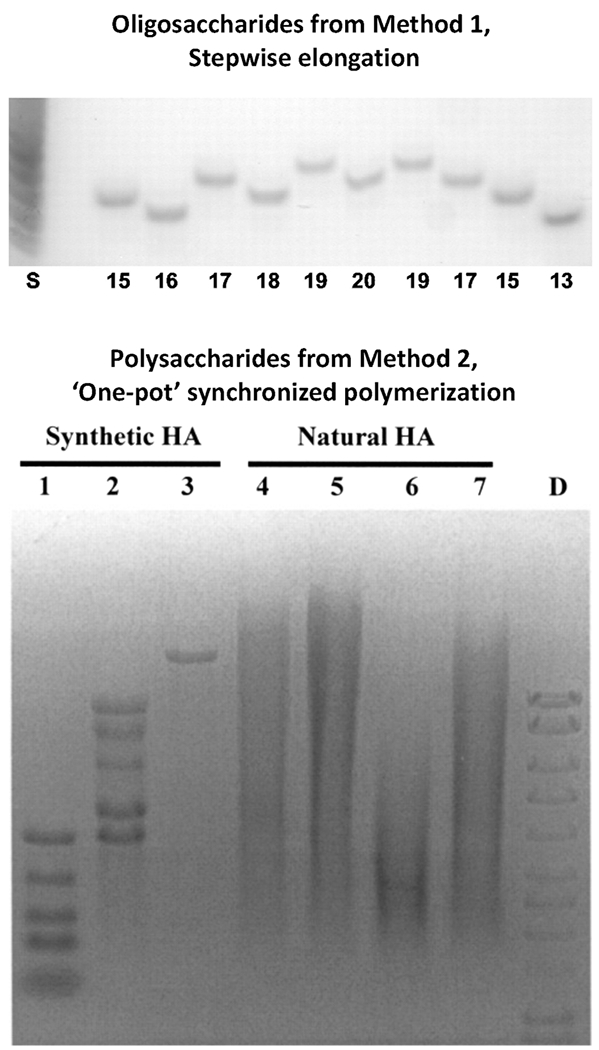
Examples of stepwise addition or synchronized polymerization reactions for authentic HA chains. *Top*. Polyacrylamide gel analysis of samples of the crude reaction mixture from the sequential sugar addition steps using immobilized monofunctional PmHAS mutants. This synthesis started with an HA tetrasaccharide and ended with an oligosaccharide comprised of 20 monosaccharide units (chain length indicated at bottom of each lane). No runaway polymerization is observed even though both UDP-sugar precursors were present at high concentration throughout the synthesis. Note that even-numbered oligosaccharides with a higher charge-to-mass ratio migrate faster than odd-numbered oligosaccharides in this system. (S, ladder of native HA digested with hyaluronidase; reproduced from *J. Biol. Chem.* (2003) 278:35199-35203) with permission). *Bottom*. Various HA samples either synthesized by synchronized chemoenzymatic reactions in vitro or isolated from streptococcal bacteria or chicken sources were analyzed on an agarose gel. Lane 1, a mixture of synthetic HA polymers produced in five different reactions (bottom to top, 27, 110, 214, 310 and 495 kDa); lane 2, a mixture of synthetic HA polymers produced in five different reactions (bottom to top, 495, 572, 966, 1090 and 1510 kDa); lane 3, 2.0-MDa synthetic HA; lane 4, rooster comb HA; lane 5, streptococcal HA (Sigma); lanes 6 and 7, streptococcal HA; D, DNA HyperLadder. The tight bands of the synthetic HA polymers, rivaling the DNA single molecular entities, indicate their relative monodispersity in comparison to natural HA. (reproduced from *J. Biol. Chem.* (2004) 279:42345-42349 with permission).

In early studies of PmHAS, two tandem GT-2 domains were observed that each contain an Asp-X-Asp motif; other GT-2s employ such motifs to coordinate the UDP-sugars and metal ion. Mutagenesis of either motif to convert the Asp residues to Asn did **not** harm the other site’s catalytic activity revealing the basis for PmHAS’s bifunctional behavior.^24^ The applied translation of this pair of monofunctional mutants, a GlcNAc-transferase (Tase) and a GlcA-transferase, led to an improved method for HA oligosaccharide production. Performing sequential, alternating step-wise additions as in Equations 3 and 4 allowed the synthesis of precisely defined HA chains^35^ (Figure 1, Figure 2 top).


(3) UDP−GlcNAc+GlcA−acceptor→GlcNAc−GlcA−acceptor+UDP

(4) UDP−GlcA+GlcNAc−acceptor→GlcA−GlcNAc−acceptor+UDP

The attachment of a monofunctional mutant catalyst (either GlcA-Tase or GlcNAc-Tase) to a bead or solid phase was a major synthetic aid; the reaction is simply allowed to go to completion before proceeding with the next extension step using the other immobilized mutant enzyme. The specific sugar unit installed at any position in the chain can be customized; for example, insertion of a NMR-active (e.g., ^13^C, ^15^N) GlcA or GlcNAc, or a non-cognate sugar with artificial structure (discussed later) are possible. The number of extension steps defines the product’s chain size. No intermediate purifications are required. As a test of catalytic control, both UDP-sugars for the entire synthesis extending HA4 to HA20 were present in the reaction mixture without issue and only de-salting was required for isolation.

Furthermore, immobilized enzymes can be readily recovered and re-used for later steps. An additional bonus of this format is that in general, bead-bound proteins including PmHAS^1-703^-derived catalysts display enhanced stability (e.g., days rather than hours) thus improving reaction economy versus use of the soluble enzymes. It should be noted that the PmCS chondroitin synthase and a *P. multocida* heparosan synthase, PmHS1, can also be used in analogous chemoenzymatic strategies to construct the structurally related glycosaminoglycans (GAGs), chondroitin or heparosan, respectively (reviewed in [15]).

### Method 2 : Synthetic HA polysaccharides via synchronized, stoichiometrically controlled polymerization reactions

Synthesis of polysaccharides (~10– 2000 kDa) requires a different strategy than Method 1 above because adding hundreds or thousands of sugar units individually would be very laborious. Fortunately, PmHAS’s nonprocessive elongation may also be used in “one-pot” synthesis format to yield HA polysaccharide with very narrow MW distribution (Table 1; Figure 1, Figure 2 bottom). Initially, it was noted that the preparations of recombinant *Escherichia coli*-derived PmHAS displayed relatively low activity in reactions in vitro containing only the two UDP-sugars and metal ion unless long incubation times (hours) were employed. However, the addition of short HA oligosaccharides (e.g., ~3–6 monosaccharide units) to the same reaction mixture boosted the incorporation activity greatly on short time scales (minutes).^20,39^

This “lag” behavior spawned the kinetic hypothesis that PmHAS^1–703^ in vitro has a slow initiation phase that first creates a short HA chain (i.e., 3–4 sugars) *de novo* from the two donors in the reaction by adding the new monosaccharide onto a UDP-sugar (or a component monosaccharide upon donor hydrolysis). In the next phase, rapid chain elongation step occurs.^42^ Overall, the use of an exogenous acceptor with 3–4 monosaccharide units acts as a “primer” allowing the PmHAS to bypass the slow initiation step in favor of the fast elongation. GlcA or GlcNAc monosaccharides, however, are **unable to** prime rapid polymerization.

Therefore, a primer will synchronize a bulk chemosynthetic reaction so that all chains are elongated simultaneously resulting in a population with very similar sizes termed quasi-monodisperse (polydispersity index values of ~1.01 or less; where “1” is the ideal polymer). While not perfect, a splay of ~5–10 major species in the size distribution of HA chains with hundreds or thousands of repeats is relatively minor for most applications. It is important to note that such narrow size distributions are **not** found in any natural polysaccharides or most synthetic polymers. Unlike DNA or proteins, there are no naturally occurring known termination signals for polysaccharides.

Furthermore, the ratio of the donor to the acceptor/primer in a reaction will control the final HA product chain size^42^ (Figures 1 and 2, Equation 5). The nonprocessive PmHAS catalyst displays a distributive nature (i.e., jumping from chain to chain in a reaction) in vitro. Therefore, for a given amount of UDP-sugars, low concentrations of acceptor will yield a few large HA polymers while the use of high amounts of acceptor yields many small chains. These reactions allow the tuning of the HA polysaccharide size to the desired target MW by simple stoichiometric control.


(5) $$n\;\text{UDP}-\text{GlcA}+n\;\text{UDP}-\text{GlcNAc}+{\left[\text{acceptor}\right]}_{x}\to 2n\;\text{UDP}+{\left[\text{GlcA}-\text{GlcNAc}\right]}_{n}-{\left[\text{acceptor}\right]}_{x}$$

Due to the distributive nature of PmHAS, even long HA chains (“megaprimers”; ~10 kDa to 1 MDa) can be extended uniformly to even larger MW products while retaining the same narrow size distribution. Therefore, complex block assembly of HA with interspersed non-HA components or segments (including chimeric GAGs discussed later) is possible. Other further structural diversification steps, if desired, can be installed by Method 1 at the junction of segments; for example, adding NMR-active isotopically-labeled native sugars or suitable unnatural sugar analogs just scratches the surface of the vast potential chemical space.

## ENHANCEMENT OF HA TARGET CHEMICAL FUNCTIONALITY VIA ACCEPTOR VARIANTS

Acceptors are key to both PmHAS chemoenzymatic synthetic strategies serving as the starting point for extension (Figure 1). While simple glucuronides are rather facile to synthesize via organic chemistry methods and widely available (e.g., GlcA-beta-*p*-nitrophenyl), it is more difficult to make shorter HA oligosaccharides via ‘green’ methods using animal-free components. Ovine (sheep) or bovine testicular hyaluronidase enzymes (which also cuts various chondroitins) were used to generate the HA oligosaccharides with GlcA residues at nonreducing terminus for the initial prototype Method 1 and 2 reactions about two decades ago. However, such products are undesirable now when preparing formulations destined for medical uses due to potential contamination issues like adventitious agents or immunogenicity/allergenicity. The recombinant human testicular HAase is available, but at significantly higher cost. Leech hyaluronidase may also be used to produce alternative HA fragments with an intact GlcNAc residue at nonreducing terminus suitable for use as a primer and is now available in high expression recombinant form.^43^

The specific *Streptomyces* HA lyase and the various chondroitin lyases (which also cut HA) enzymes are available, but these enzymes have two main problems. First, the resulting unsaturated anhydro-GlcA at the nonreducing end cannot be extended directly by PmHAS because the required hydroxyl is missing after cleavage thus an additional chemical treatment (e.g., toxic mercury reagent) is required to reveal an intact sugar residue at the nonreducing end. Second, their limit digest is a disaccharide so partial digests are needed. All of the above cleavage routes for acceptor production require intact polymeric HA as a key feedstock so that is another potential complication and expense. The use of an *E. coli* beta-1,4-GlcNAc-transferase to create the disaccharide primer from GlcA or a glucuronide as the acceptor^32^ is an alternative route to substitute for authentic oligosaccharides derived from HA digests.

The acceptor may be derivatized or constructed to possess an orthogonal functionality or useful group (e.g., amine, thiol, biotin, fluorophore, radiolabel, therapeutic agent), which becomes an integral part of the reducing terminus of **every** chain of the final chemoenzymatic product^40,42,44^ (Figure 2). This feature is a bonus of both PmHAS-based Methods 1 and 2 strategies. On the other hand, most standard subsequent chemical modifications such as reductive amination of HA termini will never modify 100% of the target molecules, especially long polysaccharides. Modification of the backbone sugars at their carboxyl or hydroxyl groups is a totally random process with respect to control of exact location and the number of coupling positions.

Another interesting, but hitherto unexplored, pathway towards HA dendrimer targets may be the use of acceptors possessing more than one GlcA-terminated moiety. Experiments comparing the synchronization proficiency of GlcA-beta- and di-GlcA- beta-fluorescein glucuronides^39^ showed that the latter was much better at priming Method 2 reactions. While not the major polymer products, both GlcA residues were observed to be extended (unpublished), so perhaps the use of better designed multivalent acceptor cores would be a path toward next-gen HA constructs with high avidity for hyaladherins and HA receptors.

As noted for the Method 1 oligosaccharide synthesis strategy above, the PmCS and PmHS1 can also be employed in a similar fashion to make the other related GAGs in a quasi-monodisperse form with potential for new reducing terminal functionalities.^15,45,46^

## NEW ROUTES TO UDP-SUGARS; *IN SITU* GENERATION AND RECYCLING SYSTEMS

UDP-GlcA and UDP-GlcNAc are the precursors for HA synthesis in vivo and in vitro. These donors were initially isolated from yeast or microbes and typically quite expensive commercially at retail (~USD$1-2 K/gram). Higher efficiency systems using recombinant cell suspensions have been described (e.g., [47]), but the use of purified UDP-sugar biosynthesis pathway enzymes in various cascades (reviewed in [48]) are cleaner, more controllable systems that can also be more amenable to incorporating unnatural monosaccharide analogs.

In brief, a GlcNAc or GlcA monosaccharide can be activated in vitro by the isolated enzymes found in the natural biosynthetic pathways that produce the UDP-sugar donors. Typically, a kinase is employed to add the phosphate group and then a uridyltransferase or UDP-pyrophosphorylase adds the UDP group. A variety of these enzymes derived from different organisms have been observed to have distinct conversion efficiencies as well as variable levels of substrate promiscuity; the latter feature drives the potential use for sugar analogs described later. For UDP-GlcA, another route employs a two-step process that first creates UDP-glucose via sucrose synthase acting in reverse with UDP, and then the action of UDP-glucose dehydrogenase to form the final product UDP-GlcA. Overall, great strides have been made for improving donor syntheses, but better catalysts and reaction formats could be beneficial.

For schema that do not require an exact set donor/acceptor ratio to precisely target a particular HA size, another advancement is the design of one-pot reactions with recycling enzyme cascade systems that convert free monosaccharides into UDP-sugars in situ for use by PmHAS without prior purification.^34,49,50^ These strategies could significantly reduce the cost of the synthesis of HA polymers as well as potentially simplify the creation of artificial sugar analogs (described in the next section) when more promiscuous pathway enzymes process the appropriate monosaccharide derivative. Another economic enhancement scheme is the use of inexpensive polyphosphate as the energy source rather than ATP.^51^

## HA ANALOGS (“HYALURONOIDS”) AND MODIFIED POLYMERS VIA UNNATURAL UDP-SUGARS

**FIGURE 3:**
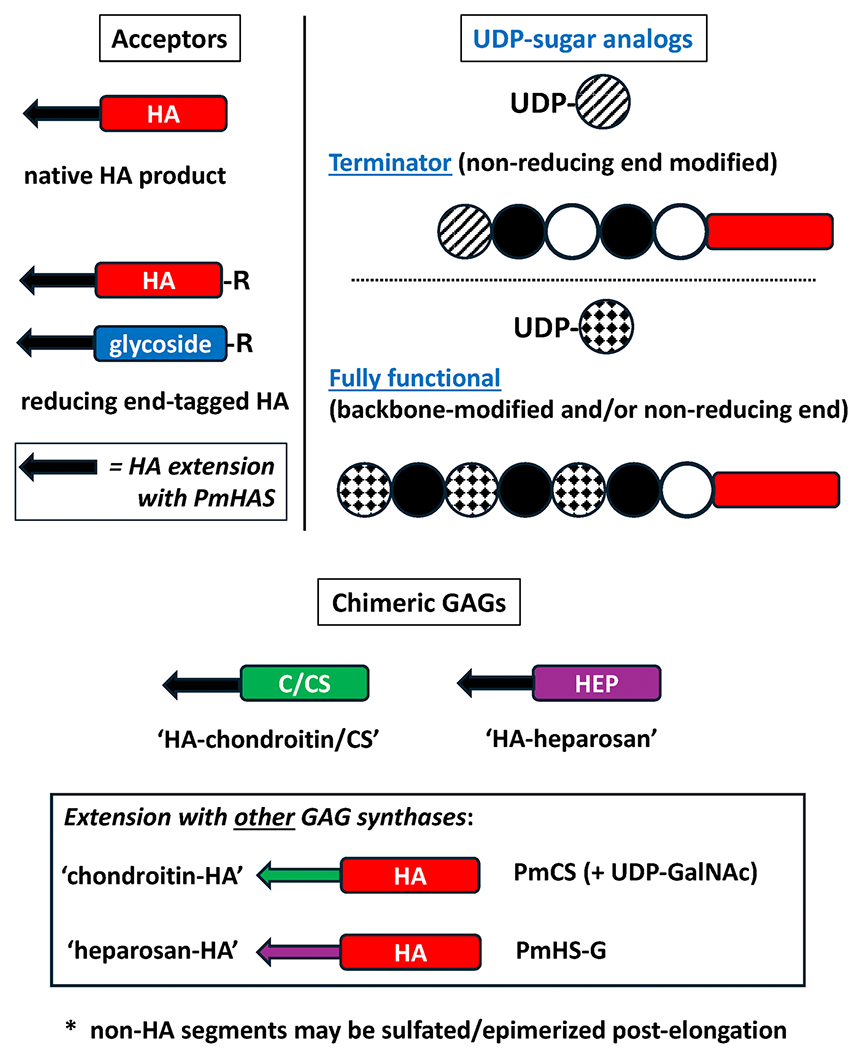
Expansion of HA carbohydrate chemical space via construction routes employing variable acceptors, UDP-sugar donor analogs, and/or alternative GAG synthases. Either of the two general chemoenzymatic methods in Figure 1 may be customized by substitution of the authentic HA substrates with alternative acceptors and/or donors. Various acceptors ranging from authentic or derivatized HA chains (*red*) or synthetic glucuronides (*blue*), to other glycosaminoglycan (e.g., chondroitin- or heparosan-based; C/Ch (*green*) or HEP (*purple*), respectively) chains may be extended with HA (*black arrow*). In addition to authentic HA precursors, UDP-GlcA and UDP-GlcNAc, various unnatural donors (sugar unit denoted with *patterned spheres*) may be used at various steps to add new chemical groups at a single or at multiple locations along the chain, depending on the donor’s incorporation properties (i.e. terminator or fully functional; Table 2). In addition to using PmHAS, other *Pasteurella* GAG synthases, PmCS and PmHS, can further diversify HA polymers into chimeric GAGs by extending an HA acceptor (oligo- or polysaccharide) with chondroitin (*green arrow*) or heparosan (*purple arrow*), respectively, provided that there is an appropriate nonreducing terminal sugar.

The HASs naturally employ the authentic donor sugars, UDP-GlcA and UDP-GlcNAc, but in some cases, PmHAS can also incorporate desirable artificial sugars into the polymer chains (Table 2, Figure 3). The ability to add new functionalities, especially chemically orthogonal groups (i.e., unique moieties beyond the HA’s intrinsic hydroxyls, carboxylates, and amides) that will not react until desired, allows further derivatization schema. While the original modification conferred by the reducing-end primer or a secondary derivative could be employed to facilitate probe generation (e.g., biotin, fluorophore, radiolabel) or drug cargo addition, and/or transform the biological properties of the polysaccharide beyond natural HA,^42^ the ability to use UDP-sugar analogs allows modification along the polymer’s backbone as well the chain’s nonreducing terminus offers new avenues to more complex polymer constructs.

Some natural biosynthetic enzymes will convert artificial monosaccharide structures to their UDP-sugar donors (reviewed in [52]). These chemoenzymatic routes offer major improvements over the total organic chemical syntheses of the past. The use of enzymes allows scalable reactions with high product yields that operate in “green” aqueous systems in the time-frame of hours; this contrasts previous multi-step, often anhydrous chemical reactions taking days with a poor yield. It has been observed that certain enzymes from some species are very promiscuous and can tolerate various non-natural functionalities on the sugar ring of the input monosaccharide albeit often at lower efficiencies than the natural substrates. This behavior has been typically discovered by empirical testing, but an exciting frontier would be the logical design and engineering of these catalysts for even better or wider analog usage.

The Class II PmHAS is the only HAS catalyst reported to date possessing the ability to use UDP-sugar analogs, but as more knowledge is gained (especially with experimentally determined or predicted polypeptide structures), perhaps the Class I enzymes may also be engineered to expand the chemical repertoire. However, the fact that the Class I’s single GT-2 domain already transfers the two different monosaccharides in two different linkages to form the HA disaccharide repeat may result in these enzymes being already limited by too many structural constraints on their main task of HA polymerization to be readily manipulated toward the expanded substrate specificity goal.

It is important to recall that for an analog to be incorporated multiple times (or to be followed by a stretch of native HA polymer), **both** of PmHAS’s GT modules need to accommodate the incoming artificial species; to start, the analog must serve as donor in the first active site, then serve as part of the acceptor (at the nonreducing terminal position) in the second active site. Then the elongated chain goes back to the first module again (where the analog resides in the penultimate position of the chain), and this challenge repeats until the artificial sugar has cleared the ‘influence zone’ of the active site or has failed.

If the UDP-sugar analog is not used as a donor by PmHAS, then it is obviously a nonfunctional substrate; PmHAS^1–703^ did not appear to incorporate UDP-GlcN[azide]^53^ and UDP-4-fluoro-GlcNAc.^54^ However, if the analog does serve as a donor, but the newly installed sugar ring is a nonfunctional acceptor, then the analog acts as a chain terminator (Figure 3, Table 2).

UDP-GlcN[trifluoroacetyl] (TFA) can be used by PmHAS to readily (~30% as efficient as authentic UDP-GlcNAc) create chains with stretches of this analog sugar.^53^ This reagent offers a protected amino group that can be readily unmasked by a post-polymerization treatment with mild base to allow further reaction with a wide variety of amine-reactive reagents including the extremely useful *N*-hydroxysuccinimides (NHS) or anhydrides. Furthermore, the native acetyl groups of HA’s GlcNAc are **not** perturbed by the deprotection step, allowing selective positional placement of free amine groups within the HA chain as desired. In this scenario, the TFA derivative would be substituted for the natural donor at any target synthesis step. This protected glucosamine is unmasked by mild base treatment and can then be reacted with NHS-esters, anhydrides, or isothiocyanates, or sulfation reagents.

However, in far more cases, the analogs were very poorly incorporated (<10% compared to authentic precursors) or were chain terminators for the native sequence PmHAS^1–703^. UDP-GlcN-alkene (for Michael addition) and UDP-GlcN-*t*Boc (acid-labile protected amine) are inefficiently incorporated by the native PmHAS under the tested conditions.^53^ The analogs UDP-4-azidoGlcNAc (useful for click chemistry), UDP-GlcN-alkyne (useful for click chemistry) and UDP-sulfoquinovose (a C6 sulfonated glucose) are chain terminators that block further extension, resulting in nonreducing end modified polymers.^53,55,56^

In a more recent innovation, UDP-4S-GlcNAc,^57^ can be used to add a free thiol group to HA’s nonreducing terminus using PmHAS, but actually a heparosan synthase chimera, PmHS-B,^58^ was a more efficient catalyst.^59^ The thiol functionality offers unique reactivity distinct from the amine and readily modified with maleimide or haloacetate reagents. It was employed to immobilize defined size HA chains via the nonreducing end to a gold surface for atomic force measurements.^59^

PmHAS appears to transfer the current analogs in the same linkage pattern as native HA [-beta-1-GlcNAc-3-beta-1-GlcA-4]*_n_*, but in the future it may be found that with some new analogs or under certain reaction conditions, the artificial sugar ring may be transferred to the polymer chain in an unnatural linkage patten. The use of the degradative enzymes (e.g., *Streptomyces* HA lyase, chondroitin AB-Case, and testicular hyaluronidase) combined with mass spectrometry or electrophoresis analysis may be generally confirmatory, but note that any observed insensitivity could also be due to the artificial sugar ring itself blocking cleavage, not an unusual linkage impeding digestion. NMR analysis, if sufficient amount of analog material is available, is currently the best experimental proof for linkage identification.

For more elaborate polymer design purposes, it is important to recall that the primer used to control chemoenzymatic synthesis (discussed above) may also be used to add an additional group to the reducing terminus thus allowing dual-tagged heterobifunctional HA chains. Obvious medical applications include directional HA linkers for drug conjugates and payload armed-biologics, or spacers for molecular assemblies. While some syntheses are possible now, the field could benefit from the rational re-design of native PmHAS to higher levels of catalytic promiscuity at improved efficiency to allow even more complex HA variant constructs.

### Chimeric glycosaminoglycans, potential mimics of matrix proteoglycans

PmHAS and PmCS can be employed to make chimeric HA/chondroitin chains (i.e., blocks of multiple sugar types) because these two glycosaminoglycans can serve as the acceptor for elongation with either synthase^41,46^ (Table 2; Figure 3). The HA and chondroitin polymers are identical except for the stereochemistry at the C4 hydroxyl of the hexosamine rings. However, the two UDP-hexosamine donors are **not** interchangeable between the two synthases; PmHAS uses UDP-GlcNAc while PmCS uses UDP-GalNAc.^30^

Several chimeric examples have been made to date including constructs in which (1) PmHAS added HA onto unsulfated chondroitin and some types of naturally sulfated chondroitin (CS), or (2) PmCS added unsulfated chondroitin onto HA. Initial studies suggest that C6-sulfated CS (CSC; shark) was elongated better by PmHAS than C4-sulfated CS (CSA; bovine)^46^ shedding some light on the synthase’s active site interaction requirements for acceptors. Chimeric octasaccharides composed of HA-chondroitin or chondroitin-HA were useful for probing the selectivity of the sugar binding interactions with hyaladherins via calorimetry and NMR.^41^

It was also observed that the PmHAS^1–703^ synthase can extend the unsulfated backbone of heparin, heparosan (HEP; [-4-GlcNAc-1-alpha-4-GlcA-1-beta-]*_n_*), to form HA-HEP chimeras if the acceptor’s nonreducing termini were GlcA residues^55^ (Figure 3). Conversely, a chimeric recombinant PmHS1/2 heparosan synthase construct, PmHS-G,^58^ will extend HA acceptors with GlcA nonreducing termini to form heparosan-HA chimeras. If not already present on a GAG chain, then the needed GlcA may be added to the nonreducing terminus using a single extension step (Method 1) and UDP-GlcA donor.

Unsulfated chondroitin and heparosan are thought to be relatively inactive molecules in the body because these “immature” unprocessed polymers are only present transiently in the Golgi and are not recognized by the hundreds of extracellular and surface proteins that require sulfate groups on these GAGs for bioactivity. Post-polymerization modification using recombinant chondroitin sulfate or heparin/heparan sulfate biosynthetic pathway enzymes (e.g., deacetylase/*N*-sulfotransferase, *O*-sulfotransferases, and C_5_-epimerases)^15^ can be employed to specifically transform the non-HA portion of chimeric products into a wide gamut of sulfated species. The current sulfation enzymes are not known to act on HA, a distinct GAG species with a different backbone structure, so this portion of the construct remains in its natural unsulfated state.

The chimeric GAGs are directly and covalently linked together via natural glycosidic bonds so these constructs would be protease-resistant and probably rather nonimmunogenic. Unlike most chemically linked structures, chimeric GAGs are predicted to be readily biodegradable in the lysosome due to their seamless sugar junctions. A vast number of chimeric GAG combinations beyond the above bipartite constructs are also possible including tripartite and even more intricate patterns.

A potential application for chimeric constructs would be to produce artificial pseudo-proteoglycan complexes **without** the proteinaceous components; while the functionality of some proteoglycans is very dependent on the features or structures of the core proteins, the interactions with the various sugar chains alone may serve well for some cell types and organs. For other systems strictly requiring these polypeptide-dependent interactions, the new chemical functionalities derived from acceptor derivatives (Figure 3) or sugar analogs (Table 2) on customized GAG chains may potentially be used to add peptides or proteins at defined sites and in known quantities.

Chimeric GAG biomaterials may be useful for tissue engineering as part of scaffolds or coatings. As GAGs and proteoglycans are very important for directing cells during development and wound healing in vivo, emulating these essential molecules with defined synthetic chimeric constructs could be a promising route to help re-create tissues and organs by guiding stem cells and grafts.

### Commercialization of HA chemoenzymatic syntheses

A wide range of factors impact the overall success of any new product ranging from economics, intellectual property, regulations, market size, user acceptance, and luck.^60^ With respect to scalability, the aspects of price and quality are key parameters of any product. For cost of goods, the recombinant PmHAS catalyst can be expressed at high levels compared to most glycosyltransferases, but improvements in enzyme yield, purification, stability, and/or specific activity should be achievable. For natural UDP-sugars, the past retail costs for isolated donors were quite high (especially considering only ~1/3 of the donor mass actually goes into the HA polymer). With the advent of harnessing the biosynthetic UDP-sugar pathway enzymes in regenerative systems to produce relatively inexpensive precursors, this factor should be much less of a hurdle in the future.

For quality, the PmHAS enzyme’s processing ability and innate characteristics in both step-wise and synchronized reaction formats is extremely favorable. For the former format, as long as each extension step is complete before proceeding to the next step, then the final HA product is relatively uniform even up to the gram level. For the latter format, as long as the donors and acceptor are thoroughly mixed before PmHAS addition, the chain size targeting and the polydispersity index are equivalent from milligram to multi-gram scales (personal observation). Furthermore, the conversion of natural UDP-sugars into HA by PmHAS can be in the 95 + % range due to its relative insensitivity to the UDP byproduct.

The need for defined HA or analog polymers made by chemoenzymatic processes will depend on their superiority over natural HAs and their derivatives for research and medicine. As HA biologists unravel the “size matters” paradigm and as new treatments and devices evolve, specialty customized polymers should be very desirable for more specific drugs targeting a bioactivity with less side effects and for biomaterials creating complex, defined architectures. Furthermore, regulatory bodies such as the U.S. Food and Drug Administration and European Medical Agency will always prefer very defined materials from non-animal or synthetic sources for human use which is a benefit of the chemoenzymatic polymer products described in this review.

## CONCLUSIONS

Overall, the PmHAS-derived toolbox has been extremely useful to synthesize various HA-based constructs for both basic and applied science goals. While the current PmHAS catalysts are powerful for production of very defined natural HA species and some variant polymers, for future challenges and needs we will need improved versions of this catalyst that can both transfer and elongate a wider variety of artificial sugars. Molecular modeling, rational design, high-throughput screening of mutants, and/or prospecting new enzyme sources should surmount this obstacle. The possibilities for synthetic strategies to produce the HA and GAG variants of the future are mainly limited by the available catalysts and our imaginations.

## Figures and Tables

**TABLE 1:** HA polymer target sizes and features of the two PmHAS-based chemoenzymatic synthesis strategies.

Method	Products
1. ***Step-wise synthesis***	Defined oligosaccharides (*n* = 1 to ~10)
2. ***Synchronized polymerization***	Quasi-monodisperse polysaccharides (*n* = ~17 to 5000)
**Shared features:** • Acceptor is installed at the reducing end of **each** product chain allowing the addition of various tags (biotin, fluorophore, etc.) and chemical functionalities (amine, *p*-nitrophenyl, etc.). • Amenable to polymers with authentic or unnatural structures. • Sugar analog addition can install nonreducing end tags (protected amine, thiol, etc.). • Block structures with multiple defined segments possible (including non-cognate GAGs).	
**Distinct features:** • Step-wise synthesis can start from a monosaccharide (typically GlcA) or its glycosides, but for the one-pot polymerization, the synchronization process requires a much better acceptor (e.g., a tri- or tetrasaccharide of HA or chondroitin) to prime efficient elongation thus form a homogenous quasi-monodisperse polymer product.	

*Note*: Authentic HA and modified/tagged variants are possible with the selection of acceptors and/or donors. The two methods can also be sequentially combined to construct more complicated multipartite polymer structures (e.g., imbedded analogs in a long chain, chimeric GAGs, etc), if desired. (*n*, disaccharide repeats).

**TABLE 2:** Artificial UDP-sugar analogs for chemoenzymatic synthesis of HA variant polymers.

Donor	Method	Analog Features	Installation position(s)
UDP-GlcN[TFA]	1 or 2	Base-labile protected amine	NR terminal or internal
UDP-4S-GlcNAc	1	Free thiol	NR terminal
UDP-sulfoquinovose	1	C6-sulfonate	NR terminal
UDP-GlcN[t-Boc]	1 or 2	Acid-labile protected amine	NR terminal or internal (low efficiency)
UDP-4-azidoGlcNAc	1	Azide click reagent	NR terminal
UDP-GlcN[alkyne]	1	-yne click reagent	NR terminal
UDP-GlcN[alkene]	1 or 2	Michael addition reagent	NR terminal or internal (low efficiency)

*Note*: Various unnatural sugars can be incorporated by wild-type PmHAS enzyme to introduce new functionalities with various utilities, however, many are not efficiently used and/or serve as a substrate for further chain polymerization.

Abbreviation: NR, nonreducing end.

## Data Availability

Overall, the data that support the findings of this review are available in PubMed and are in the public domain.

## Abbreviations:

GAG: glycosaminoglycan
GlcA: glucuronic acid
GlcNAc: *N*-acetyl-glucosamine
GT: glycosyltransferase
HA: hyaluronan
HAase: hyaluronidase
HAS: hyaluronan synthase
kDa: kiloDalton
MDa: megaDalton
MW: molecular weight
PmHAS: *Pasteurella multocida* HAS
t-Boc: tert-butyloxycarbonyl
TFA: trifluoroacetyl
UDP: uridine diphosphate

## References

[1] Meyer K, Palmer JW, J. Biol. Chem 1934, 107, 629.

[2] Simpson M, Schaefer L, Hascall V, Esko JD Essentials of Glycobiology Varki A, Cummings RD, Esko JD, Stanley P, Hart GW, Aebi M, Mohnen D, Kinoshita T, Packer NH, Prestegard JH (Eds:), Cold Spring Harbor, NY 2022, p. 205. 35536922

[3] Toole BP, J. Clin. Invest 2000, 106, 335. 10930435 10.1172/JCI10706 PMC314333

[4] DeAngelis PL, Glycobiology 2002, 12(1), 9R. 10.1093/glycob/12.1.9r 11825882

[5] Itano N, Kimata K, IUBMB Life 2002, 54(4), 195. 12512858 10.1080/15216540214929

[6] Weigel PH, DeAngelis PL, J. Biol. Chem 2007, 282, 36777. 17981795 10.1074/jbc.R700036200

[7] Weigel PH, Hascall VC, Tammi M, J. Biol. Chem 1997, 272(22), 13997. 9206724 10.1074/jbc.272.22.13997

[8] DeAngelis PL, Zimmer J, Glycobiology 2023, 33(12), 1117. 37769351 10.1093/glycob/cwad075 PMC10939387

[9] Stern R, Asari AA, Sugahara KN, Eur. J. Cell Biol 2006, 85(8), 699. 16822580 10.1016/j.ejcb.2006.05.009

[10] Cyphert JM, Trempus CS, Garantziotis S, Int. J. Cell Biol 2015, 2015, 563818. 26448754 10.1155/2015/563818 PMC4581549

[11] Tammi MI, Day AJ, Turley EA, J. Biol. Chem 2002, 277, 4581. 11717316 10.1074/jbc.R100037200

[12] Tavianatou AG, Caon I, Franchi M, Piperigkou Z, Galesso D, Karamanos NK, FEBS. J 2019, 286(15), 2883. 30724463 10.1111/febs.14777

[13] Weigel PH, Baggenstoss BA, Glycobiology 2017, 27(9), 868. 28486620 10.1093/glycob/cwx039 PMC5881711

[14] Wolny PM, Banerji S, Gounou C, Brisson AR, Day AJ, Jackson DG, Richter RP, J. Biol. Chem 2010, 285(39), 30170. 20663884 10.1074/jbc.M110.137562 PMC2943326

[15] DeAngelis PL, Liu J, Linhardt RJ, Glycobiology 2013, 23, 764. 23481097 10.1093/glycob/cwt016 PMC3671772

[16] Li J, Qiao M, Ji Y, Lin L, Zhang X, Linhardt RJ, Int. J. Biol. Macromol 2020, 152, 199. 32088231 10.1016/j.ijbiomac.2020.02.214

[17] Hagopian A, Eylar EH, Arch. Biochem. Biophys 1968, 128(2), 422. 4301574 10.1016/0003-9861(68)90048-9

[18] Blackburn MR, Hubbard C, Kiessling V, Bi Y, Kloss B, Tamm LK, Zimmer J, Glycobiology 2018, 28, 108. 29190396 10.1093/glycob/cwx096 PMC6192386

[19] Bodevin-Authelet S, Kusche-Gullberg M, Pummill PE, DeAngelis PL, Lindahl U, J. Biol. Chem 2005, 280, 8813. 15623518 10.1074/jbc.M412803200

[20] DeAngelis PL, J. Biol. Chem 1999, 274, 26557. 10473619 10.1074/jbc.274.37.26557

[21] Tlapak-Simmons VL, Baron CA, Gotschall R, Haque D, Canfield WM, Weigel PH, J. Biol. Chem 2005, 280(13), 13012. 15668242 10.1074/jbc.M409788200 PMC1592226

[22] Lombard V, Golaconda Ramulu H, Drula E, Coutinho PM, Henrissat B, Nucleic Acids Res. 2014, 42, D490. 24270786 10.1093/nar/gkt1178 PMC3965031

[23] Maloney FP, Kuklewicz J, Corey RA, Bi Y, Ho R, Mateusiak L, Pardon E, Steyaert J, Stansfeld PJ, Zimmer J, Nature 2022, 604(7904), 195. 35355017 10.1038/s41586-022-04534-2 PMC9358715

[24] Jing W, DeAngelis PL, Glycobiology 2000, 10, 883. 10988250 10.1093/glycob/10.9.883

[25] DeAngelis PL, Jing W, Drake RR, Achyuthan AM, J. Biol. Chem 1998. 273, 8454. 9525958 10.1074/jbc.273.14.8454

[26] DeAngelis PL, Papaconstantinou J, Weigel PH, J. Biol. Chem 1993, 268(26), 19181. 8366070

[27] Whitfield C, Wear SS, Sande C, Annu. Rev. Microbiol 2020, 74, 521. 32680453 10.1146/annurev-micro-011420-075607

[28] Osawa T, Sugiura N, Shimada H, Hirooka R, Tsuji A, Shirakawa T, Fukuyama K, Kimura M, Kimata K, Kakuta Y, Biochem. Biophys. Res. Commun 2009, 378, 10. 18771653 10.1016/j.bbrc.2008.08.121

[29] Pummill PE, Kane TA, Kempner ES, DeAngelis PL, Biochim. Biophys. Acta Gen. Subj 2007, 1770(2), 286. 10.1016/j.bbagen.2006.09.020 PMC1847639 17095162

[30] Jing W, DeAngelis PL, Glycobiology 2003, 13(10), 661. 12799342 10.1093/glycob/cwg085

[31] DeAngelis PL, Methods Mol. Biol 2013, 1022, 215. 23765665 10.1007/978-1-62703-465-4_17

[32] Sun JY, Deng JQ, Du RR, Xin SY, Cao YL, Lu Z, Guo XP, Wang FS, Sheng JZ, Appl. Microbiol. Biotechnol 2023, 107(16), 5119. 37405432 10.1007/s00253-023-12671-5

[33] Mandawe J, Infanzon B, Eisele A, Zaun H, Kuballa J, Davari MD, Jakob F, Elling L, Schwaneberg U, ChemBioChem 2018, 19, 1414. 29603528 10.1002/cbic.201800093

[34] Gottschalk J, Zaun H, Eisele A, Kuballa J, Elling L, Int. J. Mol. Sci 2019. 20(22), 5664. 31726754 10.3390/ijms20225664 PMC6888640

[35] DeAngelis PL, Oatman LC, Gay DF, J. Biol. Chem 2003, 278, 35199. 12840012 10.1074/jbc.M306431200

[36] He J, Huang H, Zou X, Wang Y, Du G, Kang Z, Carbohydr. Polym 2020. 231, 115700. 31888828 10.1016/j.carbpol.2019.115700

[37] Tawada A, Masa T, Oonuki Y, Watanabe A, Matsuzaki Y, Asari A, Glycobiology 2002, 12(7), 421. 12122023 10.1093/glycob/cwf048

[38] Vercruysse KP, Ziebell MR, Prestwich GD, Carbohydr. Res 1999. 318(1–4), 26. 10515049 10.1016/s0008-6215(99)00087-7

[39] Williams KJ, Halkes KM, Kamerling JP, DeAngelis PL, J. Biol. Chem 2006, 281, 5391. 16361253 10.1074/jbc.M510439200

[40] Fu X, Shang W, Wang S, Liu Y, Qu J, Chen X, Wang PG, Fang J, Chem. Commun 2017, 53, 3555. 10.1039/c6cc09431g 28286894

[41] Higman VA, Briggs DC, Mahoney DJ, Blundell CD, Sattelle BM, Dyer DP, Green DE, DeAngelis PL, Almond A, Milner CM, Day AJ, J. Biol. Chem 2014, 289(9), 5619. 24403066 10.1074/jbc.M113.542357 PMC3937638

[42] Jing W, DeAngelis PL, J. Biol. Chem 2004, 279, 42345. 15299014 10.1074/jbc.M402744200

[43] Kang Z, Zhang N, Zhang Y, Appl. Microbiol. Biotechnol 2016, 100(2), 707. 26476646 10.1007/s00253-015-7056-5

[44] Jing W, Michael Haller F, Almond A, DeAngelis PL, Anal. Biochem 2006, 355, 183. 16842731 10.1016/j.ab.2006.06.009

[45] Sismey-Ragatz AE, Green DE, Otto NJ, Rejzek M, Field RA, DeAngelis PL, J. Biol. Chem 2007, 282(39), 28321. 17627940 10.1074/jbc.M701599200

[46] Tracy BS, Avci FY, Linhardt RJ, DeAngelis PL, J. Biol. Chem 2007, 282, 337. 17099217 10.1074/jbc.M607569200 PMC4117373

[47] Meng DH, Du RR, Chen LZ, Li MT, Liu F, Hou J, Shi YK, Wang FS, Sheng JZ, Microb. Cell Fact 2019, 18(1), 118. 31262296 10.1186/s12934-019-1168-z PMC6604206

[48] Gottschalk J, Elling L, Curr. Opin. Chem. Biol 2021, 61, 71. 33271474 10.1016/j.cbpa.2020.09.008

[49] Gottschalk J, Aßmann M, Kuballa J, Elling L, ChemSusChem 2022, 15(9), e202101071. 34143936 10.1002/cssc.202101071 PMC9290584

[50] Li S, Wang S, Fu X, Liu X, Wang PG, Fang J, Carbohydr. Polym 2017, 178, 221. 29050588 10.1016/j.carbpol.2017.09.041

[51] Mahour R, Klapproth J, Rexer TFT, Schildbach A, Klamt S, Pietzsch M, Rapp E, Reichl U, J. Biotechnol 2018, 283, 120. 30044949 10.1016/j.jbiotec.2018.07.027

[52] Zhang X, Lin L, Huang H, Linhardt RJ, Acc. Chem. Res 2020, 53(2), 335. 31714740 10.1021/acs.accounts.9b00420

[53] Masuko S, Bera S, Green DE, Weiïwer M, Liu J, DeAngelis PL, Linhardt RJ, J. Org. Chem 2012, 77, 1449. 22239739 10.1021/jo202322k PMC3272127

[54] Schultz VL, Zhang X, Linkens K, Rimel J, Green DE, DeAngelis PL, Linhardt RJ, J. Org. Chem 2017, 82(4), 2243. 28128958 10.1021/acs.joc.6b02929

[55] Lane RS, St. Ange K, Zolghadr B, Liu X, Schäffer C, Linhardt RJ, DeAngelis PL, Glycobiology 2017, 27, 646. 28334971 10.1093/glycob/cwx021 PMC5458544

[56] Zhang X, Green DE, Schultz VL, Lin L, Han X, Wang R, Yaksic A, Kim SY, DeAngelis PL, Linhardt RJ, J. Org. Chem 2017, 82(18), 9910. 28813597 10.1021/acs.joc.7b01787 PMC7558457

[57] He P, Zhang X, Xia K, Green DE, Baytas S, Xu Y, Pham T, Liu J, Zhang F, Almond A, Linhardt RJ, DeAngelis PL, Nat. Commun 2022, 13(1), 7438. 36460670 10.1038/s41467-022-34788-3 PMC9718760

[58] Otto NJ, Green DE, Masuko S, Mayer A, Tanner ME, Linhardt RJ, DeAngelis PL, J. Biol. Chem 2012, 287(10), 7203. 22235128 10.1074/jbc.M111.311704 PMC3293577

[59] Giubertoni G, Pérez de Alba Ortíz A, Bano F, Zhang X, Linhardt RJ, Green DE, DeAngelis PL, Koenderink GH, Richter RP, Ensing B, Bakker HJ, Macromolecules 2021, 54(3), 1137. 33583956 10.1021/acs.macromol.0c02242 PMC7879427

[60] DeAngelis PL, Glycobiology 2021, 31(8), 886. 33822046 10.1093/glycob/cwab026

